# Cardiac MR images of thalassemia major patients with myocardial iron overload: a data note

**DOI:** 10.1186/s13104-021-05733-2

**Published:** 2021-08-19

**Authors:** Emad Shiae Ali, Mohamad Amin Bakhshali, Seyed Jafar Shoja Razavi, Hoorak Poorzand, Parvaneh Layegh

**Affiliations:** 1 Department of Medical Imaging, Alavi Hospital, School of Medicine, Mashhad University of Medical Sciences, Mashhad, Iran; 2 Department of Medical Informatics, School of Medicine, Mashhad University of Medical Sciences, Mashhad, Iran; 3 Department of Medical Imaging, Qaem Hospital, School of Medicine, Mashhad University of Medical Sciences, Mashhad, Iran; 4 Cardiovascular Department, Imam Reza Hospital, School of Medicine, Mashhad University of Medical Sciences, Mashhad, Iran; 5 Department of Radiology, Imam Reza Hospital, School of Medicine, Mashhad University of Medical Sciences, Mashhad, Iran

**Keywords:** Thalassemia major patients, Cardiac magnetic resonance imaging, Left Ventricle, Myocardial iron overload, Echocardiography

## Abstract

**Objective:**

Patients with thalassemia major (TM) have the highest mortality rate due to heart failure induced by myocardial iron overload. However, T2* weighted MR imaging is currently a gold standard approach for measuring iron overload. Examining ventricular volumes with magnetic resonance imaging (MR imaging) and measuring myocardial iron overload in TM patients allows for an early prediction of heart failure. This dataset includes cardiac MR images of TM patients and the control group with clinical and echocardiographic data. This dataset may be useful to researchers investigating myocardial iron overload. This dataset can also be used for medical image processing applications, such as ventricle segmentation.

**Data description:**

This study provides open-source cardiac MR images of 50 subjects and clinical and echocardiographic data. From February 2016 to January 2019, all images and clinical data were obtained from the MRI department of a general hospital in Mashhad, Iran. All the images are 16-bit gray-scale and stored in DICOM format. All patient-specific information is removed from image headers to preserve patient privacy. In addition, all images associated with each subject are compressed and saved in the RAR format.

## Objective

Thalassemia is a set of genetic disorders characterized by decreased or absent hemoglobin synthesis. Blood transfusion is required to control a severe form of thalassemia [1]. Blood transfusion and iron bromide absorption in the gastrointestinal tract, in either, are many times higher than normal, leading to iron overload. In patients with major thalassemia who have received blood transfusions, cardiac failure due to myocardial iron overload is the primary cause of death [2–4]. MRI uses as an appropriate modality for measuring liver and myocardial iron overload [5]. Because GRE sequences (T2* weighted) are more sensitive to tissue with iron, they are theoretically superior to spin-echo sequences for assessing myocardial iron concentration.

Because of the short imaging periods in this sequence, cardiac gated imaging with breath-holding can be used to reduce movement artifacts from heart muscle movement, blood flow, and respiratory movements [5]. Even though CMR T2* is the gold standard for detecting iron overload in myocardial muscle, diastolic cardiac dysfunction may be present even if T2* levels are normal [6]. It is crucial to assess the size and function of the ventricle in order to treat a variety of congenital and acquired cardiac diseases, including diastolic abnormalities [7]. This dataset contains CMR images and supplementary clinical data that researchers can utilize to evaluate heart function, particularly in TM patients with myocardial iron overload. They can also be used for image analysis and processing.

## Data description

This dataset consists of unenhanced Cardiac MR Images from 50 subjects, including 37 confirmed TM patients and 13 healthy subjects. Images were obtained at the point of care in an inpatient setting with echocardiographic data for heart dysfunction in TM patients and supporting clinical symptoms from February 2016 to January 2019. Subjects in this study were over the age of 18 and who showed clinical signs of heart failure (shortness of breath, decreased activity, hand, and foot swelling, round the eye and chest pain, arrhythmia), LVEF < 50%, high mean arterial pressure, renal failure, diabetes mellitus, pulmonary hypertension, addiction, cardiovascular disease and infectious diseases during the study were excluded. All CMR tests were completed a week after the last blood transfusion. All participants gave their informed consent in writing and orally.

MRI exams were performed with the Siemens Avanto B17 (1.5 Tesla) machine (Siemens Medical Solutions USA, Inc.). All images are in DICOM format and are 16-bit gray-scale images with a resolution of 192 × 256 pixels. All patient-specific information is removed from image headers to protect patient privacy. Following that, all images associated with each patient are compressed and saved in RAR format. Table 1 shows a summary of the dataset. Breath-hold and retrospective gating were applied with True FISP sequences in the short axis. A Gradient multi-echo Dark Blood sequence was used in breath-hold and retrospective gating in 3 slices in the short axis plane and 8 echo times to assess myocardial iron overload. Technically, assessment of myocardial iron overload is in the papillary muscles, the ventricular wall, and the septum. An initial slice was selected from the papillary muscles, and the other two slices were placed at the same intervals at the top and bottom of the first cut. Additional data (such as age range, sex, BSA, LAEDV index, LAESV index, LASV index, and T2*), echocardiographic data (such as LAEDV index, LAESV index, LASV index, and LAEF), and some detailed clinical data (Serum ferritin level, transfusion starting age, chelation starting age, and T2* (ms) liver iron quantification) are given as “Data file 1” in Table 1. Table 1 lists the imaging parameters for these sequences as “Data file 2.” All images were evaluated visually by two board-certified radiologists and cardiologists. Table 1, “Data file 3”, contains a complete description of the methodology.

**Table 1:** Overview of dataset

Label	Name of data file/data set	File types (file extension)	Data repository and identifier (DOI or accession number)
Data set 1	Subject (X).rar, where X = 1–37	RAR files (.rar)	Harvard Dataverse (https://doi.org/10.7910/DVN/OIWUTH) [8]
Data set 2	Subject C (X).rar, where X = 1–13	RAR files (.rar)	Harvard Dataverse (https://doi.org/10.7910/DVN/OIWUTH) [8]
Data file 1	Clinical Data.rar	RAR files (.rar)	Harvard Dataverse (https://doi.org/10.7910/DVN/OIWUTH) [8]
Data file 2	Imaging Parameters.rar	RAR files (.rar)	Harvard Dataverse (https://doi.org/10.7910/DVN/OIWUTH) [8]
Data file 3	Methodology Description.rar	RAR files (.rar)	Harvard Dataverse (https://doi.org/10.7910/DVN/OIWUTH) [8]

## Limitations

- A large number of images contain some form of motion artifacts.
- Images were taken at a single general hospital in Mashhad, Iran, representing a predominantly Iranian population.

## Data Availability

The data described in this data note can be freely and openly accessed on the Harvard Dataverse under (https://doi.org/10.7910/DVN/OIWUTH) [8]. Please see Table 1 for details and link to the data.

## Abbreviations

MRI: Magnetic resonance imaging
GRE: Gradient echo
CMR: Cardiac magnetic resonance
TM: Thalassemia major
LVEF: Left Ventricular Ejection Fraction
DICOM: Digital Imaging and Communications in Medicine
RAR: Roshal Archive
LA: Left Atrial
LV: Left Ventricle
BSA: Body Surface Area
LAEDV: Left Atrial End diastolic Volume
LAESV: Left Atrial End systolic Volume
LASV: Left Atrial Stroke Volume
LAEF: Left Atrial Ejection Fraction
